# A Method for Comparing Multivariate Time Series with Different Dimensions

**DOI:** 10.1371/journal.pone.0054201

**Published:** 2013-02-05

**Authors:** Avraam Tapinos, Pedro Mendes

**Affiliations:** 1 School of Computer Science and Manchester Institute of Biotechnology, The University of Manchester, Manchester, United Kingdom; 2 Virginia Bioinformatics Institute, Virginia Tech, Blacksburg, Virginia, United States of America; Universite de Sherbrooke, Canada

## Abstract

In many situations it is desirable to compare dynamical systems based on their behavior. Similarity of behavior often implies similarity of internal mechanisms or dependency on common extrinsic factors. While there are widely used methods for comparing univariate time series, most dynamical systems are characterized by multivariate time series. Yet, comparison of multivariate time series has been limited to cases where they share a common dimensionality. A semi-metric is a distance function that has the properties of non-negativity, symmetry and reflexivity, but not sub-additivity. Here we develop a semi-metric – SMETS – that can be used for comparing groups of time series that may have different dimensions. To demonstrate its utility, the method is applied to dynamic models of biochemical networks and to portfolios of shares. The former is an example of a case where the dependencies between system variables are known, while in the latter the system is treated (and behaves) as a black box.

## Introduction

The term ‘time series’ is used to describe a set of data points that vary over time. The analysis of different time series is an important activity in many areas of science and engineering. Methods like the Autoregressive Moving Average (ARMA) and Fourier analysis, [1] are widely used for forecasting future values based on the existing time series. Another important application is the comparison of different time series. The underlying aim of this kind of analysis is to uncover similarities and patterns that might exist in the data. This translates to four specific activities: 1) *indexing* is used to identify the most similar time series in a dataset from a query time series; 2) *classification* is used to categorize data into predefined groups [2]; 3) *clustering* is an unsupervised categorization of data [3], [4]; 4) *anomaly detection* is the identification of abnormal or unique data items [5]. For most of these activities it is necessary to compare time series using an appropriate similarity measure [6]. By similarity measure we mean any method, metric or non-metric, which compares two time series objects and returns a value that encodes how similar the two objects are. Distance metrics are commonly used similarity measures to define if two time series are similar [7].

For method *d* to be categorized as a metric, or distance metric, it must fulfill the following conditions for all *x* and *y* [8]:

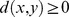
 Non-negativity

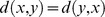
 Symmetry

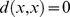
 Reflexivity

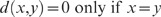
 Identity

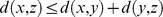
 Triangle Inequality

However, the use of metrics is not always possible or desirable. Different non-metric similarity measures provide a different perspective on comparing time series. Depending on the nature of the data one might need to use a similarity method that is not metric (does not fulfill all the distance conditions). In some cases the use of different non-metric similarity methods is more desirable since *i*) these non-metrics may be able to process data that metrics cannot and/or *ii*) provide more meaningful results than the metric methods [9], [10]. In the next section we define a semi-metric that we propose to be valuable to compare multidimensional time series.

Often it is computationally expensive (in time or storage) to apply the analysis directly to the original time series. In those cases it is more desirable to carry out the data mining analysis on shorter representations of the time series. Many methods exist for creating such representations and estimating the distance between pairs of time series approximations, such as discrete Fourier transform [11], discrete wavelet transform [12], piecewise aggregate approximation [13], or symbolic aggregate approximation [14]. These methods are widely used in many fields, including econometrics, bioinformatics and signal processing.

Of particular interest are dynamical systems composed of several variables that can be measured or simulated as a function of time. For example, models of chemical reaction networks are composed of variables representing different chemical species; stock portfolios are sets of individual stocks that are nonetheless interdependent (even though these dependencies are not known explicitly); temporal gene expression data sets represent observations of levels of different genes or gene products from an organism’s genome; models of the behavior of electronic circuits are composed of several variables that represent voltages at different points in the circuit. Up until now data mining in the context of these dynamical systems has been limited to comparisons of single time series: two particular chemical species of two biochemical models, the time series of two particular stocks, or the voltages of two points in two separate circuits. Multidimensional time series comparisons are also possible [15] but only if the various time series have the same dimensionality. These methods allow us to compare two dynamical models as long as they contain the same number of variables.

However, existing approaches [16]–[19] are not applicable when the two dynamical models have different numbers of component variables. In that case the only method that has been applied is to establish the (weighted) average behavior of each model (group of time series) and then compare the two average univariate time series [20]. While this approach may be satisfactory for some applications, it does not satisfy the needs of many others. One may be interested in comparing two groups of time series using *all* of the information contained therein, yet allowing for the two groups to have a different number of components. For example one may want to know whether a 3-variable model of calcium oscillations is more ‘similar’ to a model of calcium oscillations with 4 variables or another one with 10 variables. Equally we may want to know if the behavior of the group of 100 shares included in the Financial Times and (London) Stock Exchange (FTSE) is more similar to the group of 30 shares included in the New York Stock Exchange (NYSE) or the 50 shares included in the Shanghai Stock Exchange (SSE).

**Figure 1:**
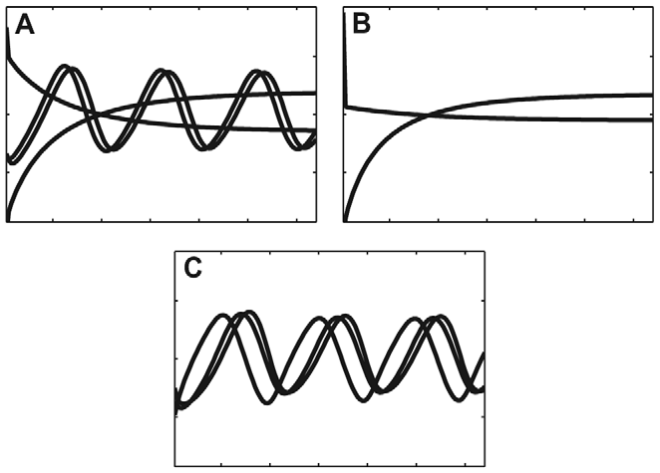
Three dynamic models with different dimensionality. **A** model with 4 variables, **B** model with 2 variables and **C** model with 3 variables. A has similarities with both B and C, however the distance between B and C is large. The question that SMETS addresses is which of B and C is closest to A?

Figure 1 illustrates the problem addressed here: three models are presented which contain different numbers of components. Clearly (and purposely) these models have some similar features: both A and C have oscillating variables with a similar frequency and relative amplitude, while both A and B have components that are monotonic. A has similarities to both B and C, but which one is ‘closer’ to or ‘more like’ A?

## Model

### Distance between Univariate Time Series

Numerous methods have been proposed for calculating the distance between univariate time series. Some of the most used are the Euclidean distance, the Manhattan distance (taxicab distance), Dynamic Time Warping (DTW), and the Longest Common Subsequence (LCSS).

Most applications in time series data mining require or benefit from some level of compression of the data since e.g. they may not fit in memory together or we may have grounds for first removing higher-frequency noise. Methods that create shorter representations of the original time series, like the Discrete Fourier Transform [11], the Discrete Wavelet Transform (DWT) [12], the Piecewise Aggregate Approximation (PAA) [13], or the Symbolic Aggregation Approximation (SAX) [14] are thus widely used. Lower bounding is a required property of these representations [21], i.e. the distance between two time series representations must be smaller or equal to the distance between the original time series. Here we use the Haar wavelet transformation method from the DWT family of representations. We then use the Euclidean distance in DWT space to measure distance between univariate time series.

### SMETS

A new method, SMETS (**S**emi **M**etric **E**nsemble **T**ime **S**eries), is proposed to compare multivariate time series of arbitrary dimensions. The method is designed to provide numerical indices that translate the level of similarity between two multivariate time series: this is achieved by matching the most similar univariate time series component between each model. The method also takes into account the differences that arise from unmatched univariate components when one of the time series has a higher dimensionality than the other.

SMETS consists of two parts: the first identifies the similarity between the two models. This is achieved by partially matching all the univariate time series components from one model (the one with the smallest number of variables) with the most similar univariate time series components from the second model. The second part of the method adds two penalties that account for the complexity of the unmatched time series and for the difference in cardinality between models. These penalties are computed from the remaining unmatched time series of the second model and the difference between the dimensions of the two time series. Consequently, the partial matching of the two models means that, in general, SMETS does not satisfy the triangle inequality rule. Since it satisfies the rest of the metric conditions (non-negativity, symmetry, identity and reflexivity), SMETS is a semi-metric method [22], [23]. In the special case where the two time series have the same dimension, then the triangle inequality is also fulfilled and SMETS is a metric.

#### Part 1, partial matching

The aim of this step is to link all the univariate time series from the model with the smallest cardinality to the most similar univariate time series from the second model. Since we are using time series representations, the distance metric used is particular to each one. The examples included here use the Haar Wavelet Transform and so the distance is simply the Euclidean distance between the DWT representations of each univariate time series. It is also possible to apply the method directly on the original time series rather than on their transformations. The partial matching proceeds according to the following algorithm:

1. Calculate the distance between each of the component time series or their representations from the model with the largest cardinality and every time series from the model with the smallest cardinality. Distances between the component (univariate) time series can be measured using any of the methods discussed above. Here the Euclidean distance in Haar DWT space is used.
2. Identify the two time series (one from each model) with the smallest distance and record that distance.
3. Remove the two component time series that were matched from further calculations.
4. Repeat steps 1 to 3 until all time series from the model with the smallest cardinality have been matched.

Two univariate time series are considered as the most similar if they share the smallest distance among all univariate time series across the two groups. Every time a pair of component time series is matched, their distance is recorded in a vector *d* and both time series are removed from the process. This step is important because it eliminates the possibility of multiple matchings of the univariate time series. Each component of the multivariate time series with the smallest dimension will therefore be matched to one and only one component of the multivariate time series with the largest dimension. Some of the components of the multivariate time series with the largest dimension will thus not be matched to a counterpart in the other multivariate time series.

After matching the most similar univariate time series, their overall distance is calculated using a *p*-norm of *d* [8] (Equation 1).
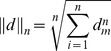
(1)


In this case *p = n*, the dimension of the smallest time series. In a set of multivariate time series, all of different dimensionality, the *p*-norm used in each comparison is different. The use of a *p*-norm here is beneficial because it provides a normalized distance value that depicts the similarity level of the partially matched time series.

The *p*-norm value calculated from Equation 1 provides an indication of the level of similarity between the matched univariate time series. However, Equation 1 does not take into consideration the influence of the unmatched component time series. Based on that, a penalty must be added to the *p*-norm to account for the dissimilarity that arises from the unmatched time series.

#### Part 2, penalization

In the second step penalties are added to account for differences between the multivariate time series. A simple way to account for the unmatched components would be to add their distance to the closest counterparts in the other multivariate time series. However it is important to account for how much information (in the sense of Shannon) is contained in the unmatched components. Thus we weight the distance between the unmatched components to the closest counterpart in the other multivariate time series by the proportion of information contained in that component. This means that unmatched time series with high information content will contribute to making the overall distance larger. Unmatched time series with little information content (e.g. constant traces) will contribute little to the overall distance. Equation 2 measures the relative information of a univariate component time series:
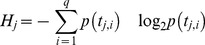
(2)


Where *H_j_* is the entropy of the (univariate) *j* component time series; *t_j,i_* is the *i*-th data point of the component time series *t_j_*; *q* is the length of the component time series, and *p(t_j,i_)* is the frequency of the value *t_j,i_* in the time series. The relative information content *RE_j_* of each unmatched component time series *j* is then:
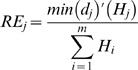
(3)


Where *d_j_* is the smallest distance between the *j*-th unmatched component time series and any component time series from the smallest model; *m* is the dimension of the larger time series. Therefore the overall entropy penalty *EP* that accounts for the distance of the unmatched components is:
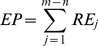
(4)


This *EP* value is then added to the *p*-norm value obtained from Equation 1.

**Figure 2:**
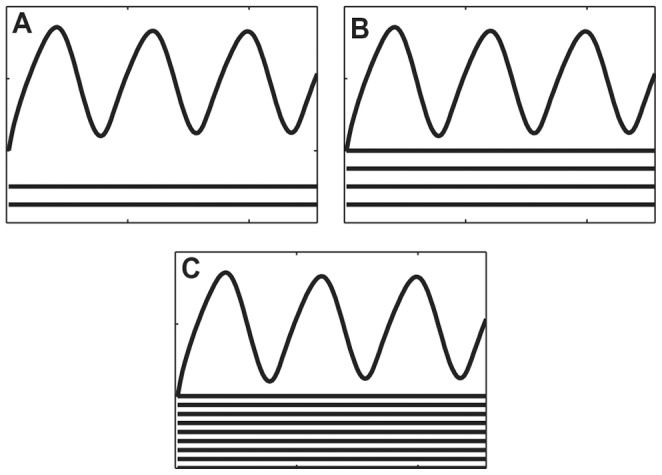
Three similar models. Models A, B and C are very similar; all three models contain an oscillating variable which behaves exactly the same and a different number of variables that are constant (zero entropy). Because SMETS also takes into account the difference of dimensions it can distinguish between these models: the distance A–B is the smallest (0.25), followed by the distance B–C (0.33) and then the distance A–C (0.54).

The *EP* penalty however would be zero if all unmatched univariate component time series were constant (since they would have zero information content), but this would violate the identity condition (see Figure 2 for an example). To avoid this and comply with the identity condition, another penalty is therefore added to account for the difference in dimensionality between the two time series. This is done through the ratio of the difference of dimensions to the sum of the dimensions:
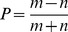
(5)


Yet, simply adding *P* to *EP* gives too much weight to the difference of dimensions and would result in that most multivariate time series of different dimension would never be similar, despite how well their components could be matched. Thus this last penalty needs to be made weaker, which is achieved with a 2-norm. Finally SMETS is described by Equation 6:
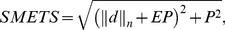
(6)which fulfills all the conditions of a semi-metric and is therefore an appropriate means for indexing multidimensional time series of arbitrary dimensions.

The reason for the addition of the second penalty (Equation 5) is best explained using the graphical example of Figure 2. Three models are presented, each of which contains a component time series with an oscillation, plus a number of other components that are static; the only difference between the models is the number of components that are static. Thus, model A has two static components, model B four, and model C nine, while all have exactly one oscillating component. Without adding the penalty of Equation 5, the distance between any pair would be exactly zero. This is the case because the unmatched components are static and therefore have zero entropy, so that in this case Equation 4 adds no penalty. However, intuitively, model C is less similar to model A than is model B because C contains a larger number of unmatched components. Equation 5 thus deals with this by taking into account the number of unmatched components. This penalty ensures the property that only objects that are exactly the same have zero distance, a requirement for semi-metrics [22], [23].

### Complexity

The time complexity of algorithms is important to ascertain whether they scale to large problems. The SMETS algorithm described here scales with the cube of the dimension of the largest time series (i.e. the one of higher dimensionality): *O*(*n* ^3^). This makes the algorithm applicable to most practical applications, even in the presence of large data sets.

## Results

To demonstrate the application of SMETS we analyze four data sets from different types of activities. The first is a financial data set of stock market financial data where SMETS is used to compare five different indices. In second place we analyze a set of time series produced from dynamic models of biochemical networks. The third data set is composed of economic data representing trade of various commodities. Finally we analyze a data set composed of electrophysiological sleep data.

### Financial Time Series Data

Financial data represent an area where SMETS seems to be well suited, as it consists largely of time series data analysis. We illustrate how it can be applied to the estimation of similarities between different stock indices. A number of stock market indices are used as benchmarks to evaluate the ‘performance’ of financial markets. Each index contains a certain number of stocks and a weighted average is usually calculated to reflect their collective performance, taken to reflect the overall performance of that market. Thus the Dow Jones Industrial Average lists 30 stocks representative of the American market, the NASDAQ-100 is an index that tracks the 100 largest non-financial companies in the National Association of Securities Dealers Automated Quotations (NASDAQ) market, the FTSE100 is an index of the 100 companies with the largest capitalization traded in the London market, the Deutscher Aktien indeX (DAX) includes 30 German companies traded in the Frankfurt market, and the SSE-50 lists the 50 major Chinese companies traded in Shanghai. Each of these can be seen as a set of connected stocks whose performance is linked (it is not important here to discuss any mechanisms of *how* they are linked), and therefore we consider their historic financial data to consist of multivariate time series. Given that each of these indices have different number of components, SMETS is appropriate to compare them. Up until now they have been compared only by the method of weighted averages (where the weights are often the relative capital of each stock). Since the weighted average destroys information, we think it may be useful to apply SMETS since this uses all of the information contained in all stocks.

Daily adjusted closing stock price data for each company represented in these indices for the period May 19, 2010 to April 18, 2011 was obtained from Yahoo Finance [24]. The data included consists of: a) FTSE 100 index and all stocks included in it have 234 data points, b) DAX 30 and all stocks included in it have 238 data points, c) Dow Jones 30 and NASDAQ 100 and all stocks included in both indices have 232 data points, d) SSE 50 and all stocks included in it have 229 data points. The differences in number of data points is due to different markets having different number of closing days (holidays, etc.).

Before applying the DWT the data were normalized by subtracting their mean value and dividing by the standard deviation. This operation is carried out on each univariate component. This normalization results in that time series are only different in their shape [25], since the differences in amplitude have been removed.

The DWT requires sequences of length that are powers of two [26]. For these data, we therefore added zeros to the end of each component time series such that the length was 256 and then transformed each one with the Haar DWT to a length 16 by keeping only the 16 coefficients with largest magnitude. In every component time series representation, the effect of zero padding affects the last symbol of the representation, so we truncated the representations to a length 15 by removing the last symbol of each one [27]. This is important to eliminate the bias that the zero padding would otherwise introduce in the comparisons.

**Figure 3:**
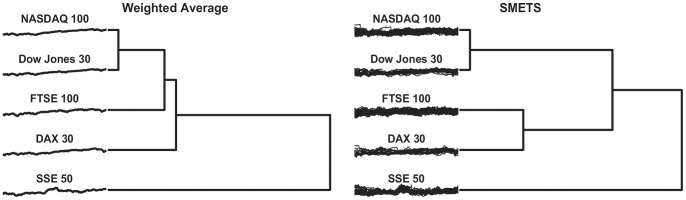
Hierarchical clustering of five stock indices. Indices were clustered based on the traditional weighted average method and on SMETS**.** The dendrogram reveals the relative distances between each entity. The time series considered by each method are represented to the left.

**Figure 4:**
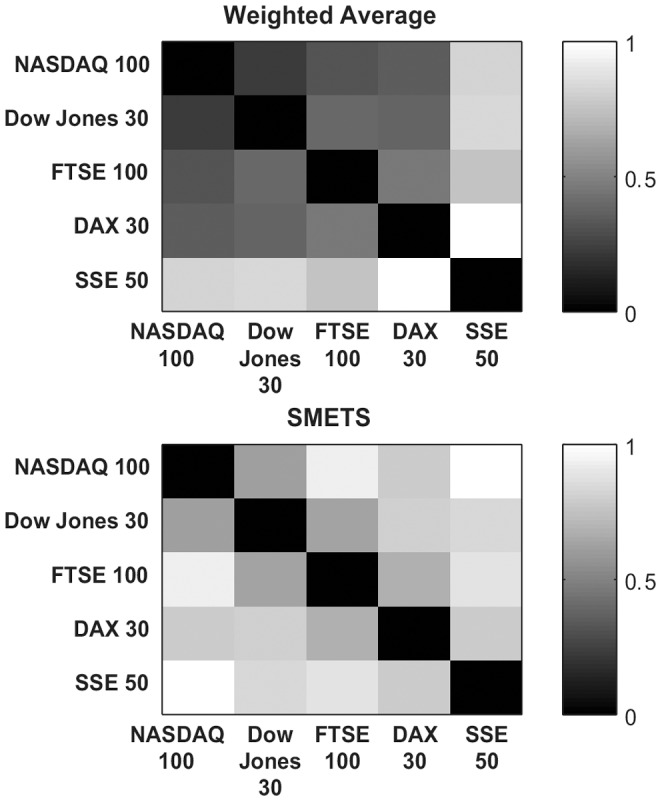
Distance matrices for the five stock indices. Distance values were measured using the weighted average and SMETS and are encoded in grayscale.

SMETS was applied to the multivariate time series for each index, which were constructed by grouping the appropriate sets of companies. A distance matrix was established based on SMETS and in parallel we used the traditional weighted averages (official indices provided by each stock market) that represent each stock (and are therefore univariate time series) and constructed a Euclidean distance matrix between them. Hierarchical clustering was applied to the two distance matrices. Figure 3 depicts the dendrograms constructed based on the clustering results that used weighted averages versus clustering results that used SMETS. The corresponding distance matrices are shown as heat maps in Figure 4. The results obtained from both the weighted average method and SMETS are not too different, however with SMETS the NASDAQ and Dow Jones indices are clearly within the same cluster, while FTSE100 and DAX group in a different one. With the weighted average method the four group within a single cluster. It is also interesting that with SMETS the FTSE100 is quite distant from the NASDAQ100. Both methods identify the SSE50 as the most dissimilar of all the indices. Plausibly these facts are related to the composition of the indices (some stocks are present in both NASDAQ100 and Dow Jones) and the nature and frequency of trades within and between specific markets.

### Biochemical Network Model Dynamics

Another area where SMETS will be useful is in modeling and simulation. Dynamical models, for example based on ordinary differential equations, represent various physical systems, such as electronic circuits or biochemical networks. Such models can be easily simulated given a certain initial condition producing time series with the behavior of the model variables. During the process of constructing and refining models it is often useful to seek other models that have similar behavior to some target. SMETS is thus well suited to this task as it allows one to find models that have some overall behavior similar to some arbitrary specification.

**Figure 5:**
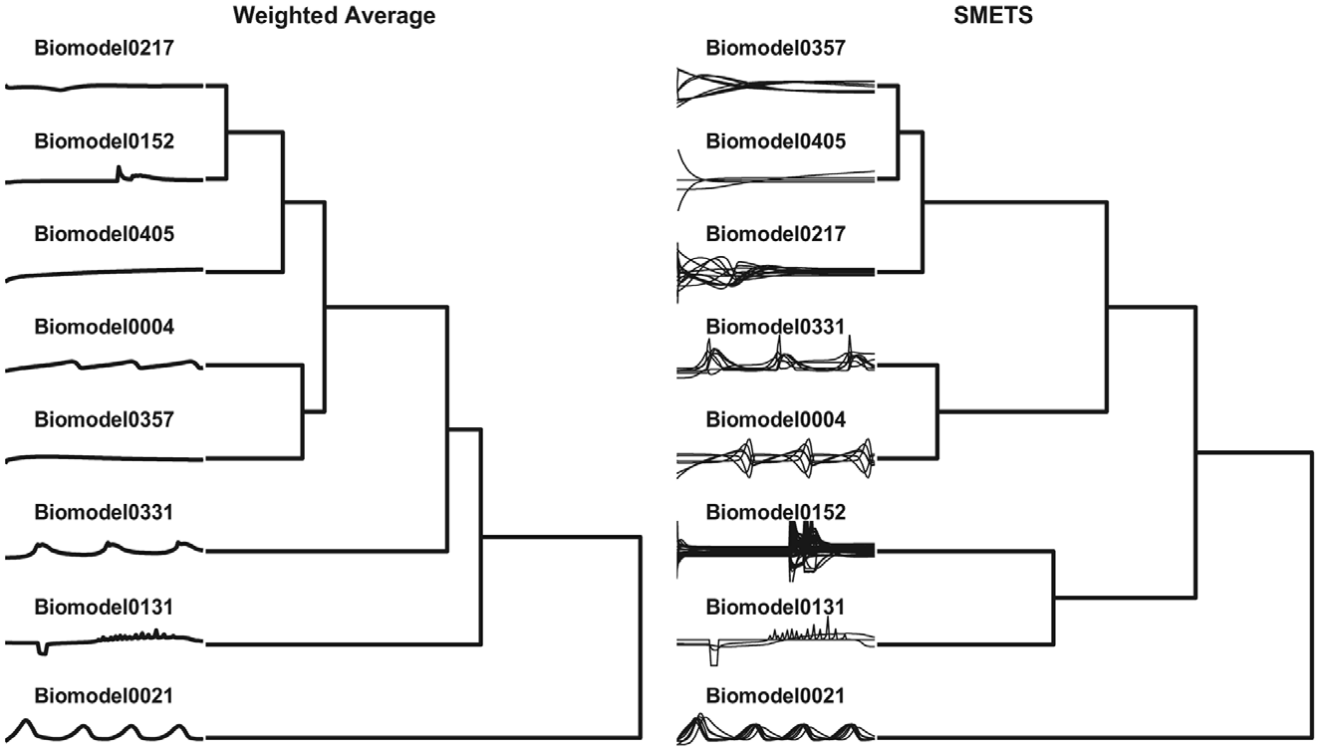
Hierarchical clustering of eight systems biology models. Models were obtained from the BioModels database [28] using average versus SMETS. The dendrogram reveals the relative distances between each entity. The time series considered by each method are represented to the left.

**Figure 6:**
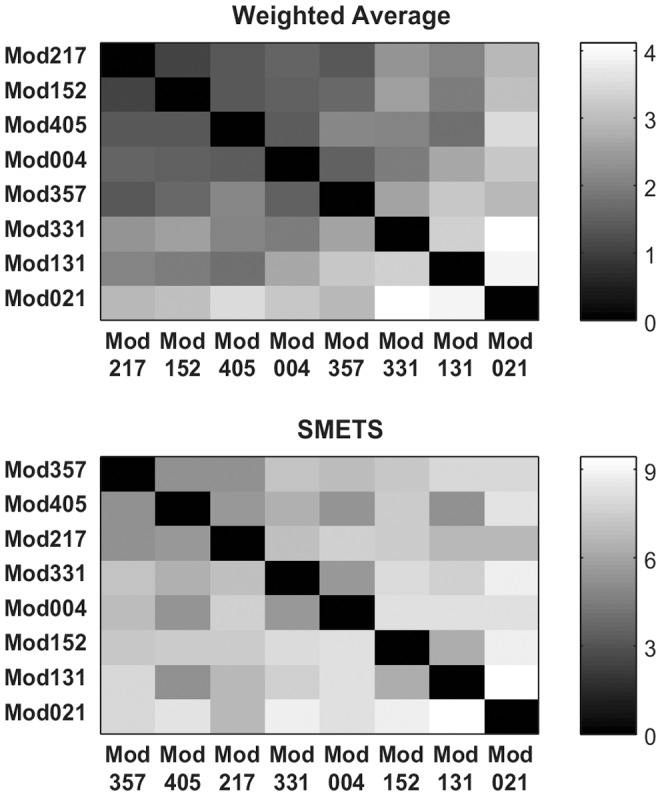
Distance matrices for the systems biology models. Distance values were measured using the average and SMETS distances and are encoded in grayscale.

In systems biology there is an initiative that is collecting all published models in a database, BioModels [28], that are made available in a standard markup language (SBML) [29]. Currently this database is indexed using a number of chemical properties of the parameters and variables in the models, but not by their behavior. It would be ideal if one could ask which model in this database behaves most similar to the one a modeler is developing. This task can be easily carried out with SMETS. To illustrate this we have extracted a small subset of eight random models from the BioModels database (models 4, 21, 131, 152, 217, 331, 357 and 405). These were then loaded into the COPASI simulator [30] which produced time series for each model by integration of their differential equations. Note that each model has a different dimension, the smallest having 3 variables and the largest 64 variables. We then applied SMETS (using the same data preprocessing as above: normalization by subtracting mean and dividing by standard deviation, followed by the Haar DWT representation using the largest 16 coefficients) to these data and used the resulting distances to establish a hierarchical clustering. In parallel we applied the average method to calculate distances that were also clustered with the same algorithm. Figure 5 depicts the classifications of the models based on each approach and Figure 6 represents the distance matrices as heat maps. It is obvious that the classification based on SMETS is different from the one based on averages. We argue that the SMETS-based classification is superior. Model 357 is clearly the most similar to 405, as identified by SMETS, however the averages method pairs it with model 4. Even qualitatively it is obvious that model 4 has sustained oscillations while model 357 does not. Model 217 is also similar to 357 and 405– its variables go through large changes in the early part of the time series and relax towards a steady state in the final part, just like the other two. But the average method pairs model 217 with model 152, yet model 152’s variables display large changes in the initial part *as well as* in the end of the time series (SMETS paired this one with model 131, which has a similar behavior)

### Economic Time Series Data

One of the main types of data studied in economics is the volume of trade of various commodities. Much like the financial data discussed earlier, these data are published both as time series of single commodities (coffee, oil, etc.) as well as weighted averages of certain groupings of commodities (energy, food, etc.). Primary commodities are a set of raw materials that can be processed and transformed to manufacture goods. Fluctuations in the price of a primary commodity can influence the price of the rest of the commodities or the prices of the final goods and have a significant influence in global economics. Therefore, different sets of primary commodities time series can be treated as multivariate time series.

The International Monetary Fund (IMF) collects the prices of primary commodities and studies the economic development of different countries. The primary commodities are categorized in groups in order to investigate the status and trends of the global economy. For each group of primary commodities’ time series a weighted average is also published that reflects the overall performance of the group.

We obtained commodity price time series data, and the group weighted averages, from the IMF website [31]. This consisted of monthly average prices of the primary commodities and the indices of different commodity groups for the period of January 2002 to August 2012. Each univariate time series has a length of 249 data points; a total of 10 groups of commodities are provided, each one having different number of component time series. Additionally some individual time series appear in more than one group, for example “bananas” appears in the following groupings: “food”, “food and beverage”, “non-fuel commodities” and “all commodities”. The groupings of the primary commodities, i.e. the multivariate time series, are: a) All Commodities, b) Non-Fuel, c) Food and Beverage, d) Food, e) Beverages, f) Industrial Input, g) Agricultural Raw Material, h) Metals, i) Energy, j) Crude Oil.

Before creating the Haar wavelet representations, each component time series was normalized by subtracting the mean value and divided by the standard deviation. Time series were padded with 7 zeros at the end of each component time series to make a length of 256. In order to eliminate bias form the zero padding, the representation was truncated to a length of 15 data points.

**Figure 7:**
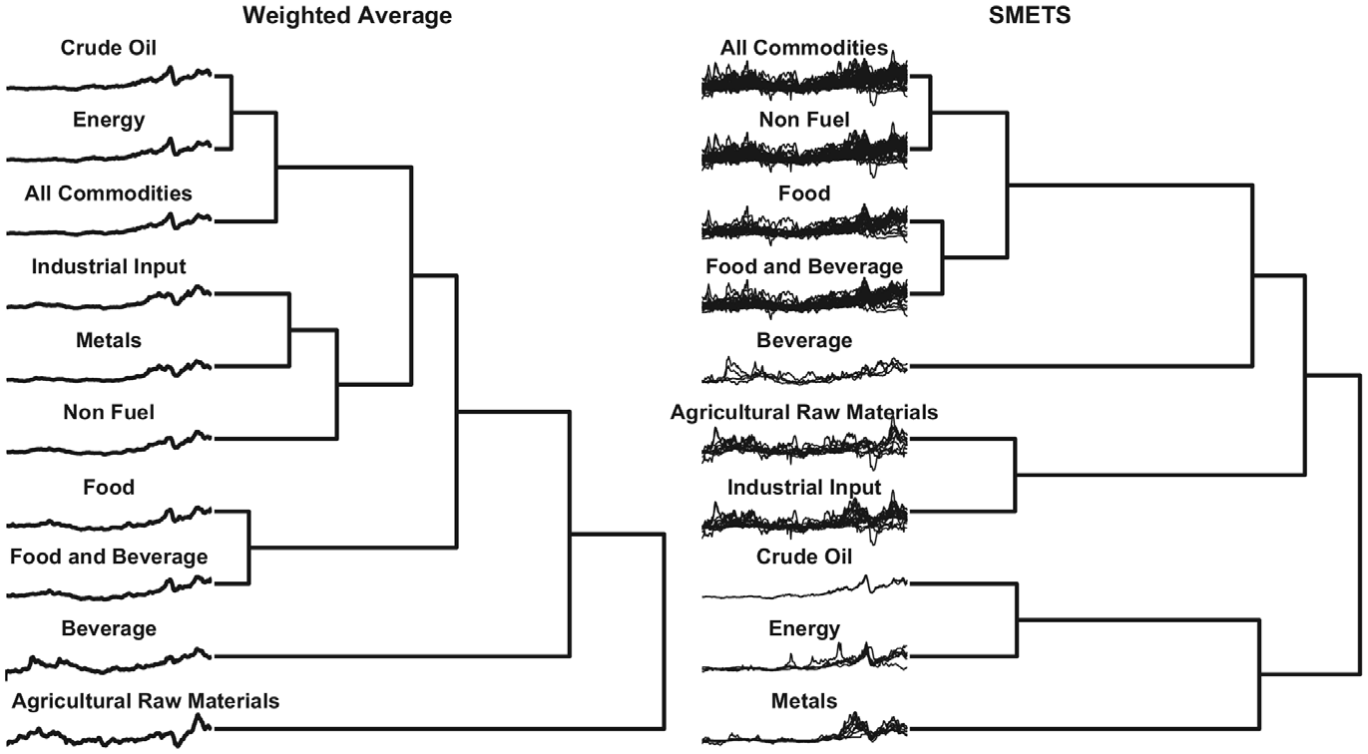
Hierarchical clustering of primary commodity prices. Distances were measured using the weighted average method versus SMETS. The dendrogram reveals the relative distances between each entity. The time series considered by each method are represented to the left.

**Figure 8:**
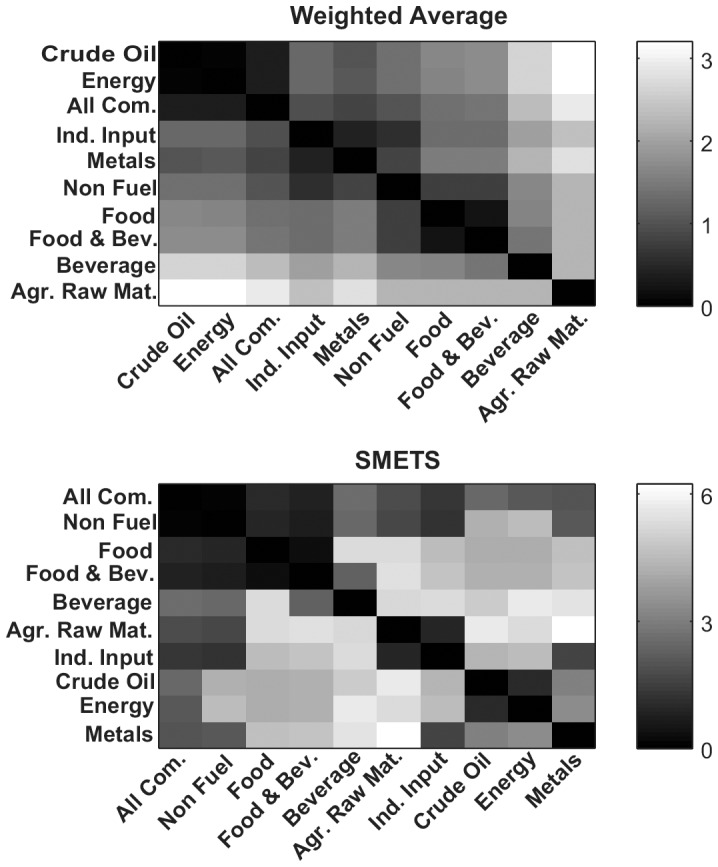
Distance matrices for the primary commodity prices. Distance values were measured using the average and SMETS distances and are encoded in grayscale.

A SMETS distance matrix was created for the different sets of commodities. In parallel, an Euclidean distance matrix was created by using the IMF indices (time series weighted averages) for comparison. Agglomerative hierarchical clustering was applied to each distance table. Figure 7 illustrates the results of hierarchical clustering in terms of dendrograms of the weighted averages and SMETS. Figure 8 depicts the distance matrices as heat maps.

The results of the two approaches are significantly different. With the classical weighted average approach the *Energy, Crude Oil* and *All Commodities* are grouped together, whereas with SMETS, *All Commodities* are clustered with *Non-Fuel commodities*. It should be noted that *All Commodities* includes all of the univariate time series that are also included in all other groups. Obviously there are common components between itself and any of *Energy*, *Crude Oil* and *Non-fuel commodities.* But there is nothing in common between *Non-fuel commodities* and either of *Energy* or *Crude Oil.* When SMETS encounters a component that is exactly equal in the two multivariate time series, it will be always matched. So the SMETS distance is smaller for the case when two multivariate time series will have the largest number of common components. In this case it is clearly *All Commodities* and *Non-Fuel commodities*, which share 45 common components. While *Energy* has only 7 in common, and *Crude Oil* only 3 in common. Because the weight of the *Crude Oil* and *Energy* components is very large, then the weighted average causes the effects of all other commodities to be minimized.

### Electrophysiological Sleep Data

Neurophysiology studies the function of the nervous system and its underlying dynamics. Various nervous system functions are investigated by means of recording and analyzing the time-dependent electric signals.

PHYSIONET [32] is a resource that gives access to many electrophysiological data sets obtained experimentally [33]. In this example sleep data from the Sleep-EDF database [34]–[35] is used. The study of sleep has identified several stages that healthy individuals go through while asleep. These studies may also provide insight into pathologies that manifest during sleep.

We obtained data from the SLEEP-EDF dataset in PHYSIONET, which consists of 8 sleep recordings, where 4 of them were obtained from healthy volunteers with no sleep difficulties [35] and the other 4 were obtained from healthy volunteers with mild difficulties in falling asleep [34]. The recordings from the volunteers with no sleep difficulties contained the following component time series EOG, FpzCz, PzOz, EEG, submental-EMG envelope, oro-nasal airflow and rectal body temperature components [35]. The recordings from the individual with the sleeping difficulties contain measurements of EOG, FpzCz, PzOz, EEG and submental-EMG envelope [34]. Thus half of the data are 7-dimensional time series, while the other half are 5-dimensional time series. Since 5 dimensions are common among all data, one could think that removing the two extra dimensions (the oro-nasal airflow and rectal body temperature, in half of the data) would provide a better classification. This is, of course, not needed for application of SMETS since it deals well with the extra dimensions. To demonstrate this, the data were analyzed in two different ways. First we apply SMETS to the unmodified data set (half of the data 7D, the other half 5D), and then we removed the 2 extra component time series in the data from normal volunteers [35] and applied SMETS to the resulting data set entirely consisting of 5-dimensional time series.

All time series were composed of 6000 data points, which were zero-padded to a length of 8192. The Haar wavelet transform was applied and the 64 largest coefficients were retained. Then the representations were truncated to a length of 47 time points (to remove the effect of zero-padding).

**Figure 9:**
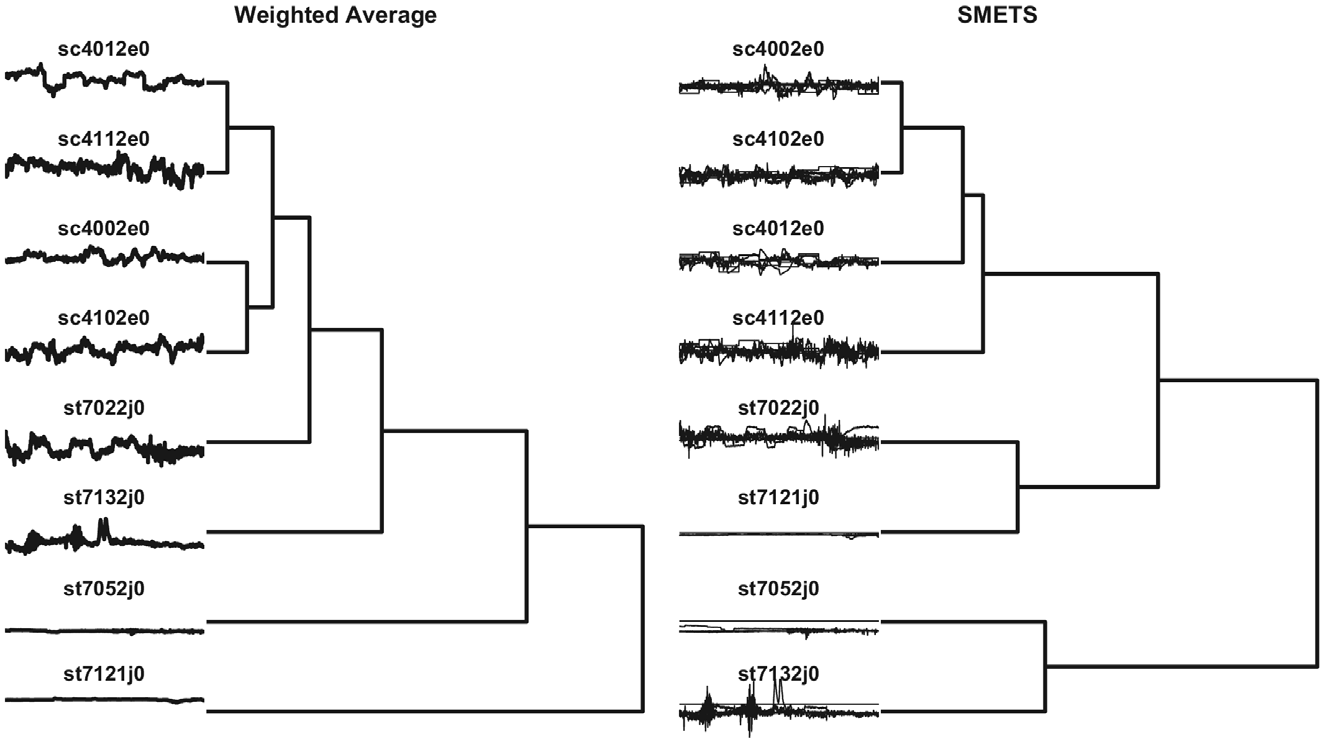
Hierarchical clustering of unmodified electrophysiological sleep data. Distances were measured using the weighted average method versus SMETS. The dendrogram reveals the relative distances between each entity. The time series considered by each method are represented to the left. Note that series sc4102e0, st7022j0 st7121j0 contain only 5 dimensions, while the other four contain 7 dimensions (see Results section for details).

**Figure 10:**
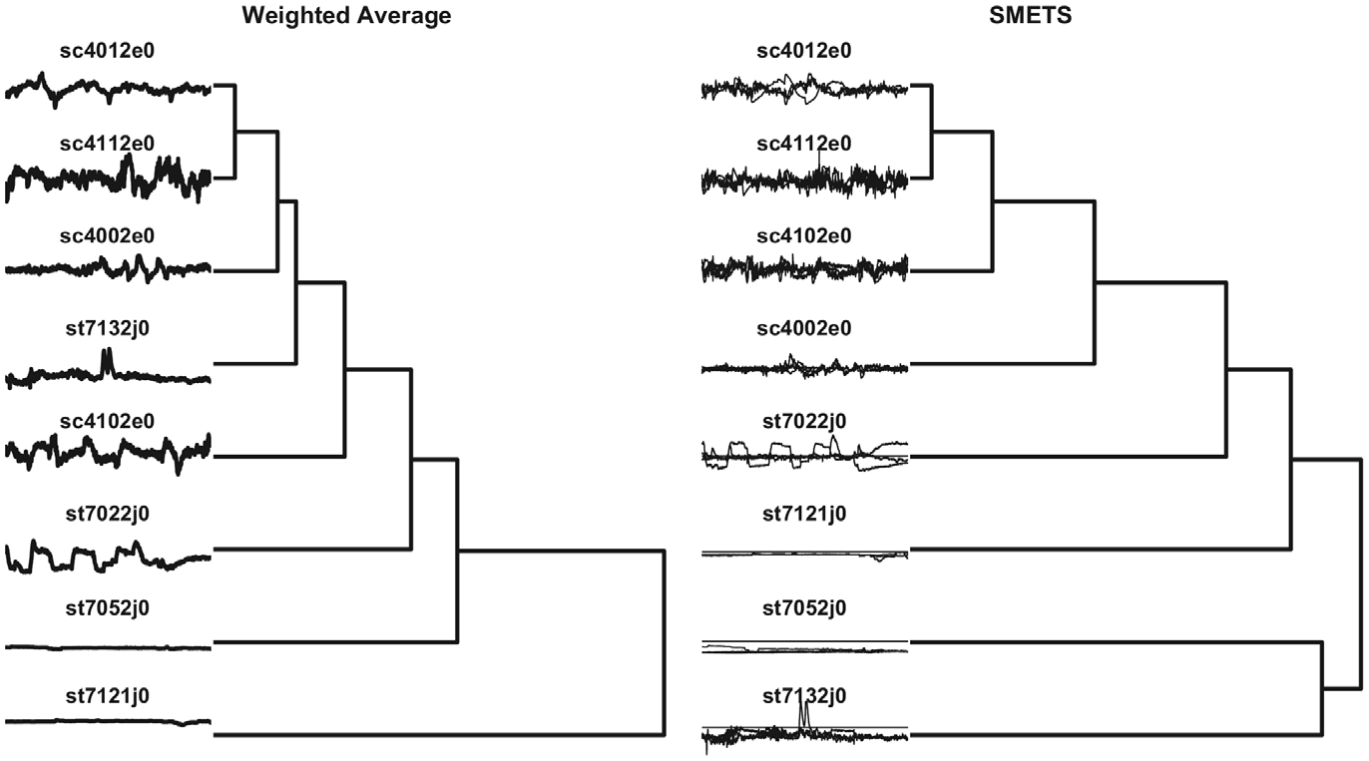
Hierarchical clustering of modified electrophysiological sleep data. Distances were measured using the weighted average method versus SMETS. The dendrogram reveals the relative distances between each entity. The time series considered by each method are represented to the left. All time series have only 5 dimensions, by removing the two extra dimensions from series sc4012e0, sc4112e0, sc4102e0 and sc4002e0 (see Results section for details).

**Figure 11:**
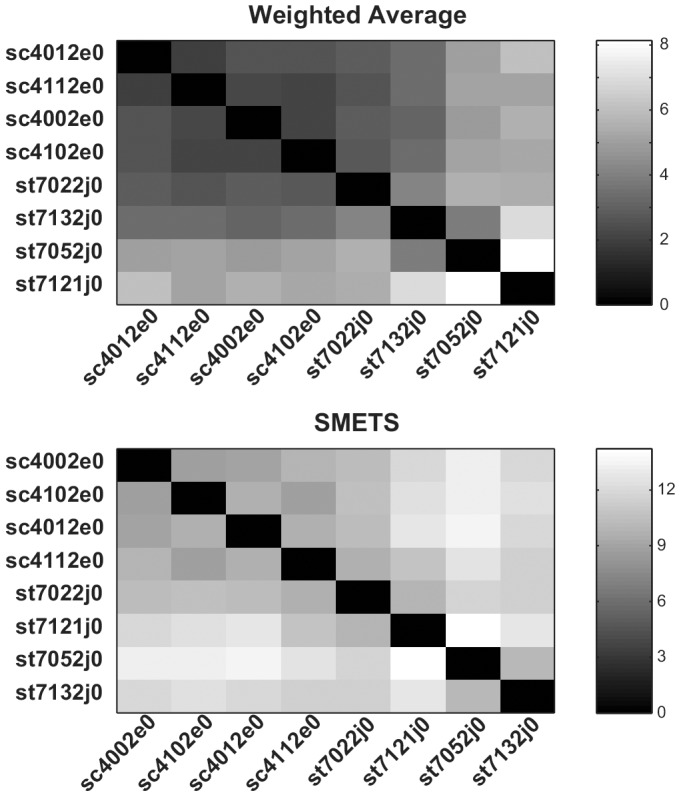
Distance matrices unmodified electrophysiological sleep data. Distance values were measured using the average and SMETS distances and are encoded in grayscale.

**Figure 12:**
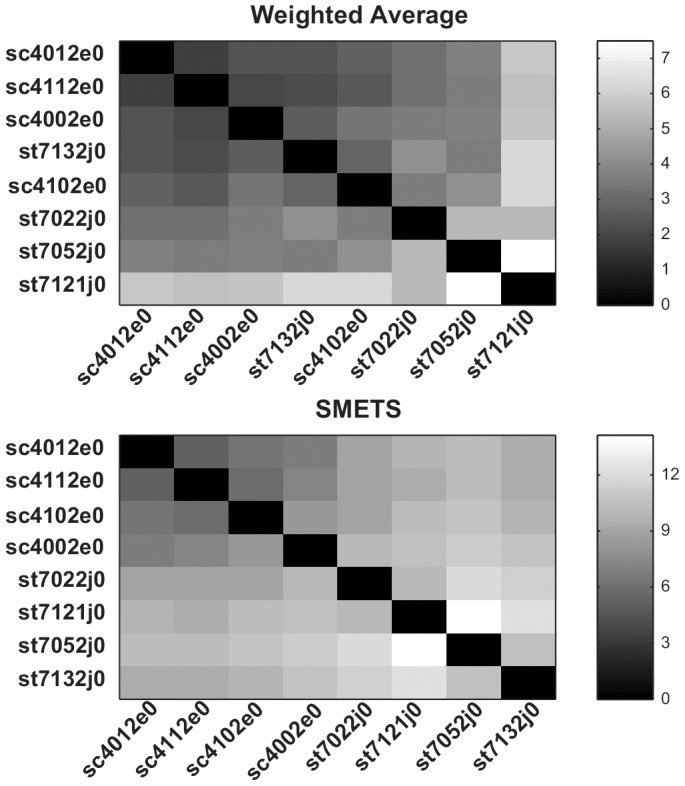
Distance matrices modified electrophysiological sleep data. Distance values were measured using the average and SMETS distances and are encoded in grayscale. Here all time series contain 5 dimensions (see Results section for details).

The resulting distance matrices obtained by applying a) Euclidean distance between the averages of all the component time series, b) SMETS applied to the unmodified data set, c) Euclidean distance between the averages of the 5 common component time series, and d) SMETS applied to a data set that was entirely composed of the 5 common component time series. Clustering of these data resulted in dedrograms depicted in Figures 9 and 10 and heat maps in Figure 11, and 12.

The results are not too different with any of the four methods; essentially all cluster the normal individuals together. In the complete data set (7D/5D) SMETS shows a better separation between normal individuals and those with sleep problems. However it is possible that this is the result of the bias introduced by the difference of dimensions (because all normals are 7D and all sleep disorders 5D). To remove this possible bias in the data, we eliminated the extra two components in the data of normal individuals. In this case both the averages method and SMETS show a somewhat less demarked separation. But clearly both methods still are capable of separating normals from disorders.

## Discussion

We propose a method – SMETS – for comparing multivariate time series with different dimensionalities. It calculates the distance between the most similar components of two multivariate time series, and then adds penalty values to account for the difference in their dimensionalities. The penalty value is calculated using Shannon’s entropy of the unmatched components. Thus, SMETS uses all of the information contained in both time series, despite their different dimensionality, which makes this method unique.

Current methods for comparing multivariate time series like the Euclidean distance, dynamic time warping [16], weighted sum singular value decomposition (WSSVD) [17], principal component analysis similarity factor (SPCA) [18] and extended Frobenius norm (EROS) [19] are limited to applications where the time series are of equal dimensionality. SMETS removes this restriction and allows distances to be calculated even when the data are of different dimensions. The examples presented demonstrate that SMETS can identify similarities without being too influenced by the difference in dimensions. A distinctive example is the case of the behavior of two biological models from the BioModels database: Model 131 contains only 3 variables while model 152 contains 64 variables, yet despite this large difference, their SMETS distance is small, allowing them to cluster together (Figures 5 and 6). This is entirely justified because both models display similar temporal behavior: variables from both models change rapidly in the initial stage and then again towards the end of the observation, while in between they have little variation. By contrast, the traditional weighted average obscures their similarity.

Both the financial and biological model examples reveal an advantage of using SMETS over the weighted averages method. Averaging all of the component time series destroys a great deal of information but SMETS avoids this and uses all of the data contained in all components. The matched components all contribute to the calculation of similarity, while the unmatched components add a penalty to the distance. Both Figures 3 and 5 show cases where the original multivariate time series are very different, but the average of their components is similar. This is especially obvious in the biological models example where even visual inspection (Figure 5) shows that the classification is more accurate with SMETS. For example the BioModels 217 and 152 have a similar average behavior but are quite distinct when considering all their component time series. This is less clear in the dendrograms of the financial data, probably because those time series are quite similar to start with (i.e. the stocks included in those indices are strongly correlated). However both distance matrices, when viewed as heat maps (Figures 4 and 6), show that SMETS reveals more structure in the data than method of averages.

The example with economic data presents an interesting case where some component time series are common between multivariate time series. This is because the classes are hierarchical and, for example the component *West_Texas_Intermdiate* belongs to *Crude Oil*, as well as to *Energy* and to *All Commodities*. When applying SMETS these components are guaranteed to be matched. The SMETS analysis puts emphasis on the similarity of time-dependent patterns, whereas the IMF weighted average puts more emphasis on commodities that have large trades. The result is that with SMETS *All Commodities* is closer to *Non Fuel Commodities* while with the IMF weighted averaging *All Commodities* is closer to *Crude Oil* and *Energy*. If the objective of the comparison is to find what part of the economy has the largest weight then the weighted averages is the most suitable. On the other hand, SMETS is best to identify which multivariate time series are most similar based on their time dependent patterns.

One of the growing trends in data mining is the use of very large data sets (sometimes known as “big data”). Searching for patterns in such datasets is often hard due to their size and dimensionality. SMETS is applicable to such datasets because it can easily be combined with time series representations that compress the data by orders of magnitude. In the examples above we used a wavelet transform representation and the distance calculations were carried out in that space, allowing for the full time series to be discarded as only the representations are needed for calculations.

SMETS is, to our knowledge, the only method that allows comparing multivariate time series of different dimensionality that uses all of the information contained therein. Therefore we propose that SMETS will be a useful tool for time series data mining.
